# Acidophil stem cell-like pituitary neuroendocrine tumors with heterogeneous PIT1/SF1 or LH lineage-marker expression: a clinicopathological study of three cases

**DOI:** 10.3389/fendo.2026.1879290

**Published:** 2026-07-23

**Authors:** Hong Zheng, Xue Yang, Meili Zhang, Weiyi Chen

**Affiliations:** 1 Department of Pathology, Qilu Hospital of Shandong University Dezhou Hospital, Dezhou, Shandong, China; 2 Department of Pathology, Affiliated Yantai Yuhuangding Hospital of Qingdao University, Yantai, Shandong, China

**Keywords:** 2022 WHO classification, acidophil stem cell-like pituitary neuroendocrine tumor, heterogeneous lineage-marker expression, PIT1, serial-section mapping, SF1

## Abstract

Acidophil stem cell-like pituitary neuroendocrine tumors are uncommon PIT1-lineage tumors with heterogeneous morphology and immunophenotypes. Their distinction from other PIT1-lineage tumors and from tumors with additional gonadotroph-lineage-marker expression remains challenging. We retrospectively analyzed three surgically resected acidophil stem cell-like PitNETs. Clinical, endocrine, radiological, operative, histological, immunohistochemical, and follow-up data were reviewed. In cases 2 and 3, spatially matched serial sections were used to assess the relationship between PIT1 and SF1 expression. All patients were male and aged 17–48 years. The tumors showed PIT1 and PRL expression with variable GH expression. Case 1 showed LH positivity but was SF1-negative. Cases 2 and 3 showed SF1 positivity but were negative for FSH and LH. Spatially matched serial sections in cases 2 and 3 demonstrated PIT1-positive and SF1-positive nuclei in morphologically corresponding tumor-cell areas across consecutive serial sections, supporting PIT1/SF1 lineage-marker co-expression. All tumors showed radiological and/or intraoperative evidence of local invasiveness. The Knosp grades were bilateral grade 3 in case 1, grade 4 in case 2, and grade 1 in case 3. Early postoperative MRI showed no visible residual tumor in all cases. At the last available MRI follow-up, no recurrence or progression was identified after 14.7, 51.3, and 57.6 months in cases 1, 2, and 3, respectively. Acidophil stem cell-like PitNETs may show heterogeneous PIT1/SF1 or LH lineage-marker expression. Spatially matched serial-section analysis supports PIT1/SF1 co-expression in a subset of tumors; however, the biological significance and classification implications of this pattern require further study. Integrated interpretation of morphology, transcription factors, hormones, cytokeratin pattern, endocrine findings, radiological invasiveness, and longitudinal follow-up is essential.

## 1 Introduction

Pituitary neuroendocrine tumors (PitNETs) are common neoplasms of the sellar region. In recent years, with the incorporation of pituitary transcription factors into routine pathological diagnosis, the classification of PitNET has gradually shifted from a traditional system based predominantly on hormone expression to an integrated, cell lineage-based framework. The 2022 World Health Organization (WHO) classification of endocrine and neuroendocrine tumors emphasizes the combined assessment of transcription factors, pituitary hormones, and morphological features for PitNET subtyping. In this system, PIT1, SF1, and TPIT correspond to distinct adenohypophyseal cell differentiation lineages, and acidophil stem cell PitNET is classified among PIT1-lineage tumors (1).

Acidophil stem cell PitNET is an uncommon PIT1-lineage tumor that usually expresses prolactin (PRL) and may show variable growth hormone (GH) expression (2). Its classic ultrastructural hallmark is the presence of giant mitochondria in the cytoplasm. Previous studies have suggested that this tumor is clinically associated with hyperprolactinemia; some patients may present with mild or atypical manifestations of acromegaly, whereas serum GH levels may remain normal or only minimally elevated (3). Compared with conventional lactotroph adenomas, acidophil stem cell tumors may be larger and more invasive and may show serum PRL levels that are disproportionate to tumor volume (4).

In diagnostic practice, however, acidophil stem cell PitNET overlaps to some extent with mixed somatotroph-lactotroph tumors, mammosomatotroph tumors, mature or immature PIT1-lineage plurihormonal tumors, and multilineage PitNET. When a tumor simultaneously expresses PIT1-lineage markers and gonadotroph-lineage-related markers such as SF1 and LH/FSH, its precise classification and biological implications remain incompletely defined. Both the 2022 WHO classification and recent practice-oriented diagnostic recommendations emphasize that PitNET diagnosis should integrate transcription factors, hormone-expression profiles, cytokeratin-staining patterns, clinical endocrine manifestations, and radiological invasiveness, rather than relying on a single hormone immunophenotype (5).

In the present study, we collected three cases of acidophil stem cell-like PitNET. All patients were male and ranged in age from 17 to 48 years. Their clinical presentations included visual acuity and visual field impairment, cerebrospinal fluid rhinorrhea, and headache. All three tumors showed PRL expression with variable GH expression, and some cases also expressed gonadotroph-lineage-associated markers, including SF1 or LH, suggesting immunophenotypic heterogeneity. By integrating clinical manifestations, imaging features, histomorphology, immunohistochemical findings, and the relevant literature, this study analyzes the diagnostic criteria, classification, differential diagnosis, and biological behavior of these tumors, with the aim of improving recognition of acidophil stem cell-like PitNETs and their heterogeneous lineage-marker expression patterns.

## 2 Materials and methods

### 2.1 Case selection

Three surgically resected cases pathologically diagnosed as PitNET between January 2021 and December 2025 were collected. All three patients were male, aged 17–48 years, with a median age of 34 years. Case 1 was from Shandong University Qilu Hospital Dezhou Hospital, and cases 2 and 3 were from Yantai Yuhuangding Hospital Affiliated to Qingdao University. All cases involved sellar mass lesions, and all patients underwent tumor resection via an endoscopic or navigation-assisted transsphenoidal approach. The inclusion criteria were as follows: (1) complete clinical and imaging data; (2) a lesion located in the sellar or suprasellar region, with histological specimens obtained by surgery; (3) an immunophenotype compatible with acidophil-lineage differentiation, characterized by PIT1 and PRL expression with variable GH expression; and (4) in some cases, simultaneous expression of gonadotroph-lineage-related markers such as SF1 or LH, requiring re-evaluation of lineage assignment under the 2022 WHO classification system. Metastatic tumors, craniopharyngiomas, Rathke cleft cysts, germ cell tumors, and other non-pituitary sellar lesions were excluded.

The patients’ age, sex, major clinical manifestations, past medical history, preoperative endocrine test results, imaging findings, surgical approach, intraoperative observations, pathological diagnosis, immunohistochemical results, and postoperative follow-up data were retrospectively collected. Clinical endocrine data focused on serum PRL, GH, adrenocorticotropic hormone (ACTH), cortisol, thyroid function, gonadal-axis-related hormones, and postoperative pituitary function. Imaging assessment focused on tumor size, suprasellar extension, cavernous sinus invasion, internal carotid artery encasement, sphenoid sinus or sellar floor bone invasion, optic chiasm compression, and Knosp grade.

All case data were anonymized. This study was reviewed and approved by the ethics committees of the participating institutions. Case 1, from Shandong University Qilu Hospital Dezhou Hospital, was approved under the simplified ethics review procedure of Shandong University Qilu Hospital Dezhou Hospital (approval/application No. 202504271013000429051). Cases 2 and 3 from Yantai Yuhuangding Hospital Affiliated to Qingdao University were approved by the Ethics Committee of Yantai Yuhuangding Hospital, with the Approval No. 2025-193. The requirement for informed consent was waived by the respective ethics committees because of the retrospective design and anonymized use of clinical data.

### 2.2 Histological evaluation

All surgical specimens were fixed in 10% neutral buffered formalin, routinely dehydrated, paraffin-embedded, serially sectioned at 4 μm, and stained with hematoxylin and eosin. Two senior pathologists jointly reviewed the slides without reference to the original diagnostic conclusions. The evaluation included tumor architecture, cellular morphology, eosinophilic or chromophobic cytoplasmic change, cytoplasmic vacuolation, mitotic activity, necrosis, hemorrhage, fibrosis, and the relationship with surrounding tissues. Particular attention was paid to morphological features suggestive of acidophil stem cell-like PitNET, including eosinophilic cytoplasm, oncocytic-like change, cytoplasmic vacuolation or granularity, focal chromophobic areas, and whether cytokeratin staining showed a perinuclear dot-like or fibrous-body-like pattern.

### 2.3 Immunohistochemistry

Immunohistochemical staining was performed using the EnVision two-step method or an automated immunohistochemistry platform. All primary antibodies were commercially available ready-to-use antibodies. PIT1, SF1, TPIT, PRL, ACTH, TSH, CK8/18, and SSTR2 were purchased from Fuzhou Maixin Biotech Co., Ltd.; GH, FSH, and LH were purchased from Xiamen Talent Biomedical Technology Co., Ltd.; Syn, CgA, p53, and Ki-67 were purchased from Beijing Zhongshan Golden Bridge Biotechnology Co., Ltd.; and ER was purchased from Roche Diagnostics. All antibodies were used according to the manufacturers’ instructions. Antigen retrieval was performed using high-temperature heat-induced retrieval in EDTA buffer, high-temperature heat-induced retrieval in citrate buffer, or the corresponding automated-platform protocol.

The extent of immunoreactivity was recorded as the approximate percentage of positive tumor cells after review of representative tumor areas. Staining intensity was graded as weak, moderate, or strong. Nuclear localization was recorded for PIT1, SF1, TPIT, and ER, whereas cytoplasmic or membranous localization was recorded for pituitary hormones, cytokeratins, and SSTR2.

### 2.4 Spatially matched serial-section mapping

For cases 2 and 3, spatially matched consecutive 4-μm serial sections were prepared from the same paraffin block and stained for PIT1 and SF1. Corresponding tumor areas were identified using stable histological landmarks, including tumor-cell nests, vascular structures, stromal septa, and nuclear morphology. The sections were independently reviewed by two pathologists to determine whether PIT1-positive and SF1-positive signals were present in morphologically corresponding tumor-cell areas.

In case 1, consecutive serial sections stained for PIT1 and LH were reviewed using the same approach. This analysis was used to assess spatial correspondence of lineage-marker expression across serial sections; it was not a dual-label immunohistochemical assay.

## 3 Results

### 3.1 General clinical characteristics and endocrine phenotypes

The cohort comprised three male patients aged 17–48 years, with a mean age of 33.0 years. The initial clinical symptoms in all cases were related to tumor mass effect or involvement of local structures rather than to a typical hormone-hypersecretion syndrome. Case 1 was a 17-year-old male who presented with progressive visual deterioration and visual field defects. He had no clinical manifestations of acromegaly, lip or tongue enlargement, polydipsia, or polyuria. Case 2 was a 34-year-old male who developed watery or lightly blood-stained nasal discharge that worsened with head lowering, bending, and lateral decubitus after the discovery of a pituitary tumor; cerebrospinal fluid rhinorrhea was clinically suspected. His initial serum PRL concentration was reported as >470.0 ng/mL before bromocriptine therapy. Case 3 was a 48-year-old male who presented with headache accompanied by nausea and vomiting. He had no hyposexuality, polydipsia, polyuria, acromegaly, body-weight change, or centripetal obesity; preoperative PRL was 504.9 ng/mL.

The detailed preoperative, early postoperative, and last available follow-up endocrine data are summarized in **Supplementary Table 2**. In case 1, preoperative PRL and GH levels were 30.82 ng/mL and 1.336 ng/mL, respectively. In case 2, the initial preoperative PRL concentration was reported as >470.0 ng/mL before bromocriptine therapy; the PRL level of 111.6 ng/mL was obtained after dopamine-agonist treatment. In case 3, preoperative PRL was 504.9 ng/mL. Preoperative endocrine profiles, including GH, ACTH, cortisol, thyroid-axis hormones, LH, FSH, testosterone, and corresponding laboratory reference ranges, are provided in **Supplementary Table 2**.

None of the patients had overt clinical manifestations of acromegaly. However, IGF-1 was not measured in any case; therefore, the absence of a clinical acromegalic phenotype should not be interpreted as biochemical exclusion of GH excess. The cases showed variable PRL elevation and incomplete endocrine information at some time points, emphasizing the importance of integrated clinical, biochemical, radiological, and pathological assessment.

### 3.2 Imaging and intraoperative features of invasiveness

All three tumors were located in the sellar region and showed varying degrees of local invasiveness. Case 1 had the largest tumor. MRI revealed an intrasellar and suprasellar mass measuring approximately 6.0×4.5×3.8cm. The lesion extended upward through the diaphragma sellae, forming a “snowman” configuration, compressed the optic chiasm, and extended beyond the lateral tangent lines of the bilateral internal carotid artery C2-C4 segments, consistent with bilateral Knosp grade 3 involvement. Intraoperatively, the tumor protruded into the sphenoid sinus, invaded the sellar floor bone, involved both cavernous sinuses, adhered tightly to the internal carotid arteries in some areas, and extended toward the anterior aspect of the optic chiasm and the floor of the third ventricle. A high-flow cerebrospinal fluid leak occurred during surgery.

In case 2, initial MRI showed a sellar mass measuring approximately 2.5×2.1×3.4cm, with inferior growth, sellar floor depression, sphenoid sinus compression, and encasement of both internal carotid arteries, more prominently on the left. After oral bromocriptine therapy, repeat MRI showed tumor shrinkage with partial cystic change, and a water-like signal was observed in the sphenoid sinus. In conjunction with the clinical presentation, cerebrospinal fluid rhinorrhea was considered. Intraoperatively, the sellar floor bone was thin and invaded by tumor. The intrasellar tumor was friable and “fish-flesh-like,” with relatively rich vascularity and abundant fibrous strands and septa. Approximately 3×3×2cm of tumor was removed. On retrospective MRI review, the maximum Knosp grade was 4.

In case 3, MRI demonstrated an intrasellar lesion measuring approximately 2.6×1.9×1.6cm with right parasellar/cavernous sinus extension, corresponding to Knosp grade 1. Intraoperatively, the sellar floor bone was thin and invaded by tumor. Gray-white intrasellar tumor tissue with relatively rich vascularity was identified, and most of the tumor was aspirated. The resected tissue measured approximately 3×3×2cm.

**Figure 1:**
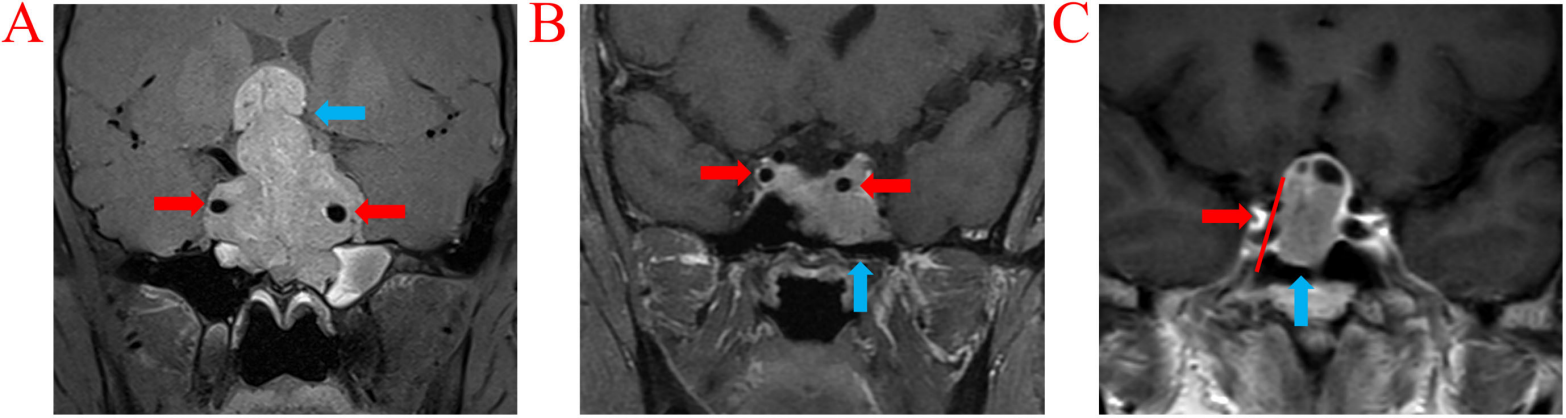
Radiological assessment of local invasiveness and Knosp grading. **(A)** In Case 1, a giant sellar/suprasellar mass with a characteristic “snowman” configuration is observed (blue arrow). The lesion extends bilaterally beyond the lateral tangent lines of the C2–C4 segments of the internal carotid arteries (red arrows). The lesion was classified as Knosp grade 3. **(B)** In Case 2, the tumor shows inferior extension with sphenoid-sinus compression and fluid-like signal within the sphenoid sinus (blue arrows), together with bilateral internal carotid artery encasement (red arrows). The lesion was classified as Knosp grade 4. **(C)** In Case 3, the tumor extends beyond the medial tangent line of the right internal carotid artery but does not cross the intercarotid center line (red arrow). Inferior extension of the tumor is also observed, with compression of the sphenoid sinus (blue arrow). The lesion was classified as Knosp grade 1.

Thus, although the clinical endocrine manifestations in these three cases were atypical, all tumors showed local invasiveness confirmed by imaging or intraoperative findings, including sellar-floor invasion, cavernous sinus extension, internal carotid artery encasement, sphenoid-sinus invasion in case 1, and sphenoid-sinus compression or fluid-like signal in cases 2 and 3 (**Figure 1**). The clinicoradiological, endocrine, surgical, and short-term postoperative features of the three cases are summarized in **Supplementary Table 1**.

### 3.3 Histological features and initial pathological diagnoses

**Figure 2:**
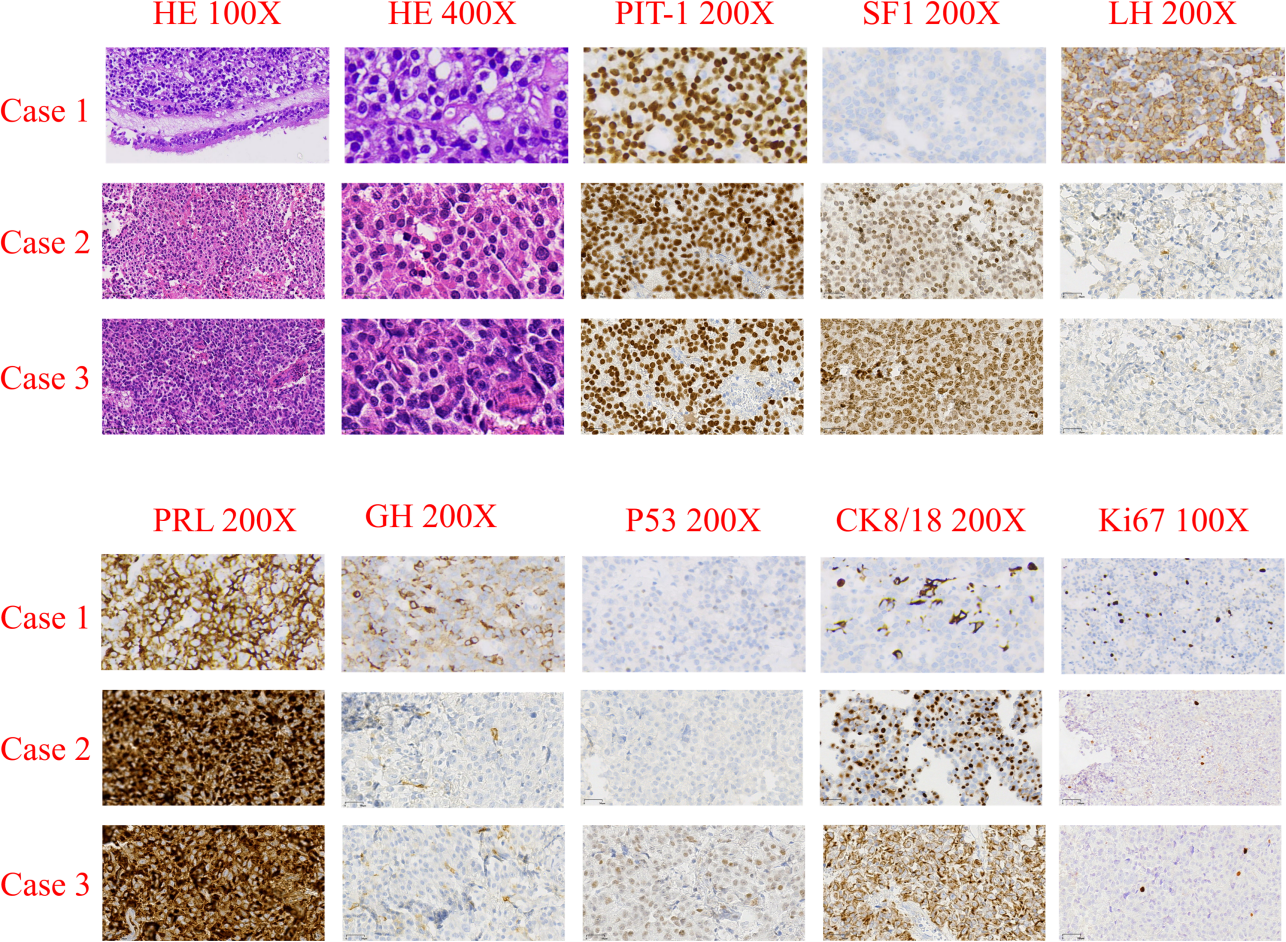
Histological and immunohistochemical features of the three tumors. Representative H&E sections and immunostains from cases 1-3. H&E images show PitNET morphology at x100 and x400. Immunohistochemistry demonstrates nuclear PIT1 expression and PRL expression with variable/focal GH expression in all cases. Case 1 was SF1-negative but showed LH immunoreactivity, whereas cases 2 and 3 showed SF1 nuclear positivity without definite LH expression. p53, CK8/18, and Ki-67 staining are shown for ancillary assessment; The Ki-67 labeling indices were approximately 5%–10%, 1%, and 2% in cases 1, 2, and 3, respectively. Original magnifications are indicated above each column. Abbreviations: H&E, hematoxylin and eosin; PIT1, pituitary-specific transcription factor 1; SF1, steroidogenic factor 1; LH, luteinizing hormone; PRL, prolactin; GH, growth hormone; CK8/18, cytokeratin 8/18.

All three tumors were resected through an endoscopic transsphenoidal approach. On hematoxylin-eosin staining, all tumors exhibited PitNET morphology. Tumor cells were arranged in sheets, nests, and trabeculae, with abundant stromal blood sinusoids; focal pseudopapillary or gland-like structures were also observed. The tumor cells were medium-sized and relatively uniform, with indistinct cell borders and abundant eosinophilic to granular cytoplasm. Some tumor cells showed pale, vacuolated, or chromophobic cytoplasmic changes, resulting in a heterogeneous cytological appearance. The nuclei were round to oval, with finely granular chromatin and occasional small nucleoli. Overall nuclear atypia was not prominent. In some areas, the cytoplasm was condensed, showing oncocytic-like change; focally, tumor cells were arranged around vessels or fibrous stroma. No extensive necrosis was identified, and mitoses were rare. In individual cases, tumor tissue infiltrated surrounding fibrous tissue, bone, or submucosal tissue, consistent with the local invasiveness suggested by imaging (**Figure 2**). The postoperative pathology of case 1, in combination with immunohistochemistry, primarily suggested acidophil stem cell adenoma, with mixed GH-PRL adenoma as a differential diagnosis; this case also showed LH expression and a relatively high Ki-67 proliferation index. Case 2 was diagnosed as pituitary adenoma, and immunohistochemistry supported a plurihormonal adenoma expressing acidophil-cell-lineage and gonadotroph-cell-lineage markers. Case 3 was diagnosed as pituitary adenoma; in combination with the immunohistochemical results, the findings were consistent with a plurihormonal adenoma expressing gonadotroph-cell-lineage and acidophil-cell-lineage markers.

Electron microscopy was not available for any case; therefore, classic ultrastructural features emphasized in acidophil stem cell tumors, such as giant mitochondria, could not be evaluated. Accordingly, based on the shared PRL expression, focal or variable GH expression, CK-positive pattern, and ER expression in some cases, the term “acidophil stem cell-like PitNET” is used in this study. The diagnostic boundaries between these tumors and PIT1-lineage plurihormonal PitNETs or multilineage PitNETs under the 2022 WHO classification are further analyzed below.

### 3.4 Transcription-factor, hormone, and cytokeratin immunophenotypes

All three tumors showed a shared PIT1-lineage immunophenotypic background, with diffuse strong nuclear PIT1 expression in 100% of tumor cells and strong PRL expression in most tumor cells. GH expression was variable among the cases: case 1 showed strong immunoreactivity in approximately 50% of tumor cells, whereas cases 2 and 3 showed moderate immunoreactivity in approximately 10% of tumor cells. ACTH, TSH, TPIT, and FSH were negative in all three cases.

Case 1 showed an immunophenotype characterized by diffuse PIT1 expression, strong PRL expression, and substantial GH expression, together with strong cytoplasmic LH immunoreactivity in approximately 90% of tumor cells. In contrast, SF1 and ER were negative. This profile supports PIT1-lineage differentiation with concurrent LH lineage-marker expression, rather than a conventional SF1-lineage gonadotroph PitNET. The Ki-67 labeling index was approximately 5%–10%. Spatially matched serial sections in case 1 also showed PIT1-positive and LH-positive signals in morphologically corresponding tumor-cell areas, supporting spatial association of PIT1 and LH expression within the same tumor-cell population.

Cases 2 and 3 both showed diffuse strong nuclear expression of PIT1 and SF1, together with strong PRL expression and focal GH expression. In both cases, FSH and LH were negative. To determine whether PIT1 and SF1 expression represented spatially separate tumor components or co-expression within the same tumor-cell population, spatially matched serial-section mapping was performed. Consecutive 4-μm sections from the same paraffin block were stained for PIT1 and SF1, and corresponding tumor regions were identified using stable histological landmarks, including tumor-cell nests, vascular structures, fibrous septa, and nuclear morphology. In both cases, PIT1-positive and SF1-positive nuclei were identified in morphologically corresponding tumor cells within the same tumor-cell nests. These findings provide spatial evidence supporting PIT1/SF1 lineage-marker co-expression within the same tumor-cell population and argue against a simple admixture of spatially separate PIT1-lineage and SF1-lineage tumor components. Representative serial sections are shown in **Supplementary Figure 1**.

The cytokeratin staining patterns were heterogeneous. In case 1, CK8 immunoreactivity was detected in approximately 2% of tumor cells. In case 2, CK8/18 showed paranuclear dot-like positivity in approximately 80% of tumor cells. In case 3, CK8/18 showed strong membranous immunoreactivity in essentially all tumor cells. ER was negative in case 1, showed moderate nuclear positivity in approximately 60% of tumor cells in case 2, and showed strong nuclear positivity in all tumor cells in case 3. SSTR2 was strongly positive in approximately 90% of tumor cells in case 1 but was negative in cases 2 and 3.

The Ki-67 labeling indices were approximately 5%–10%, 1%, and 2% in cases 1, 2, and 3, respectively. Overall, the immunophenotypic findings support acidophil stem cell-like PIT1-lineage differentiation in all three tumors, with heterogeneous additional lineage-marker expression: LH expression without SF1 expression in case 1 and spatially supported PIT1/SF1 lineage-marker co-expression in cases 2 and 3. The detailed histopathological and immunohistochemical findings are summarized in **Table 1**.

**Table 1:** Histopathological and immunohistochemical findings.

Feature	Case 1	Case 2	Case 3
Specimen/gross description	Gray-white to gray-red fragmented tissue, approximately 2.5 x 2.0 x 0.7 cm	Small gray-white tissue fragment, approximately 0.8 x 0.4 x 0.2 cm	Gray-white to gray-yellow irregular tissue fragments, approximately 2.0 x 2.5 x 0.7 cm
Histological pattern	PitNET morphology; sheets/nests/trabeculae with rich sinusoidal vasculature; eosinophilic or granular cytoplasm with focal pale/chromophobic/vacuolated change	PitNET morphology; plurihormonal adenoma with acidophil-cell-lineage and gonadotroph-lineage marker expression	PitNET morphology; plurihormonal adenoma with gonadotroph-cell-lineage and acidophil-cell-lineage marker expression
Original/pathology-report interpretation	Acidophil stem-cell adenoma was favored; mixed GH-PRL adenoma was listed as a differential diagnosis; concurrent LH expression and relatively high Ki-67 were noted	Pituitary adenoma; immunophenotype supported a plurihormonal adenoma expressing acidophil-cell-lineage and gonadotroph-cell-lineage markers	Pituitary adenoma; immunophenotype supported a plurihormonal adenoma expressing gonadotroph-cell-lineage and acidophil-cell-lineage markers
PIT1	100%; strong; nuclear	100%; strong; nuclear	100%; strong; nuclear
SF1	0%; negative	100%; strong; nuclear	100%; strong; nuclear
TPIT	0%; negative	0%; negative	0%; negative
PRL	90%; strong; cytoplasmic	100%; strong; cytoplasmic	100%; strong; cytoplasmic
GH	50%; strong; cytoplasmic	10%; moderate; cytoplasmic	10%; moderate; cytoplasmic
ACTH	0%; negative	0%; negative	0%; negative
TSH	0%; negative	0%; negative	0%; negative
FSH	0%; negative	0%; negative	0%; negative
LH	90%; strong; cytoplasmic	0%; negative	0%; negative
CK8/18	2%; cytoplasmic	80%; paranuclear dot-like positivity	100%; strong; cytoplasmic
P53	5%; moderate	1%; moderate	80%; moderate
ER	0%; negative	60%; moderate; nuclear	100%; strong; nuclear
SSTR2	90%; strong; membranous	0%; negative	0%; negative
Ki-67 index	Approximately 5%-10%	Approximately 1%	Approximately 2%

### 3.5 Lineage-expression patterns and diagnostic boundaries

Based on the combined morphological and immunohistochemical findings, all three tumors showed a PIT1-lineage background, characterized by diffuse PIT1 expression, strong PRL expression, and variable GH expression. Together with the eosinophilic or granular cytoplasmic features and heterogeneous CK8/CK8/18 staining patterns, these findings support acidophil stem cell-like differentiation. However, because electron microscopy was not available to demonstrate the characteristic giant mitochondrial accumulation of classic acidophil stem cell PitNET, the designation “acidophil stem cell-like PitNET” is considered more appropriate than a definitive diagnosis of classic acidophil stem cell PitNET.

Despite their shared PIT1/PRL/GH background, the three tumors showed distinct additional lineage-marker expression patterns. Case 1 demonstrated diffuse PIT1 expression, strong PRL expression, substantial GH expression, and strong cytoplasmic LH expression in approximately 90% of tumor cells, whereas SF1 and FSH were negative. This pattern supports a PIT1-lineage tumor with concurrent LH expression. Although the strong and diffuse LH positivity indicates that this finding is unlikely to represent merely rare entrapped non-neoplastic cells, the absence of SF1 and FSH does not provide sufficient evidence for conventional gonadotroph-lineage differentiation. Therefore, case 1 is best interpreted as an acidophil stem cell-like PIT1-lineage PitNET with heterogeneous hormone expression, rather than as a conventional gonadotroph PitNET.

Cases 2 and 3 both showed diffuse strong nuclear expression of PIT1 and SF1, together with strong PRL expression and focal GH expression, while FSH and LH were negative. Spatially matched serial-section mapping demonstrated PIT1-positive and SF1-positive nuclei in morphologically corresponding tumor cells within the same tumor-cell nests in both cases. These findings support PIT1/SF1 lineage-marker co-expression within the same tumor-cell population and make a simple admixture of spatially separate PIT1-lineage and SF1-lineage tumor components less likely. Nevertheless, the lack of FSH and LH expression indicates that the SF1-positive phenotype did not show a complete gonadotroph hormone-expression profile.

Accordingly, cases 2 and 3 are best described as acidophil stem cell-like PIT1-lineage PitNETs with spatially supported PIT1/SF1 lineage-marker co-expression. This wording recognizes the observed co-expression pattern while avoiding the assumption that these tumors represent a fully established, biologically distinct multilineage PitNET subtype. Overall, the present cohort represents a heterogeneous group of acidophil stem cell-like PitNETs, including one tumor with LH expression in the absence of SF1 expression and two tumors with PIT1/SF1 lineage-marker co-expression. The diagnostic interpretation of each case should therefore integrate morphology, transcription-factor profile, hormone expression, cytokeratin pattern, endocrine findings, radiological behavior, and spatial assessment of lineage-marker expression.

### 3.6 Postoperative outcomes and follow-up

Short-term postoperative recovery was generally acceptable in all three patients. Case 1 experienced postoperative agitation and fever and required management for a high-flow cerebrospinal fluid leak, which improved after continuous lumbar cistern drainage and anti-infective therapy. At discharge, bilateral visual acuity had improved markedly compared with the preoperative status, and no obvious visual field defect remained; prednisone replacement therapy was prescribed because of low pituitary hormone levels. Case 2 had no persistent nasal or nasopharyngeal fluid leakage after surgery. He received anti-infective therapy for fever and increased cerebrospinal fluid leukocytes and was discharged after the cerebrospinal fluid leukocyte count decreased on repeat testing; after discharge, he continued oral bromocriptine and regular follow-up. Case 3 had no postoperative cerebrospinal fluid rhinorrhea or obvious polydipsia/polyuria, no significant electrolyte abnormalities, and was discharged after vital signs stabilized.

Early postoperative MRI demonstrated no visible residual tumor in all three cases. At the last available MRI follow-up, no radiological recurrence or progression was observed in case 1 on March 8, 2026, corresponding to 14.7 months after surgery; in case 2 on July 12, 2025, corresponding to 51.3 months after surgery; and in case 3 on November 10, 2025, corresponding to 57.6 months after surgery. Follow-up included sellar MRI and repeat endocrine testing. These observations should be interpreted cautiously because the follow-up duration was substantially shorter in case 1.

## 4 Discussion

### 4.1 Shift in the diagnostic logic of PitNETs under the 2022 WHO classification

The 2022 WHO classification of endocrine and neuroendocrine tumors further emphasizes that PitNETs should be diagnosed according to adenohypophyseal cell lineage rather than solely on the basis of hormone immunoexpression or clinical endocrine syndromes. PIT1, SF1, and TPIT correspond to the PIT1 lineage, gonadotroph lineage, and corticotroph lineage, respectively. The PIT1 lineage encompasses somatotroph, lactotroph, and thyrotroph differentiation. The PIT1-lineage category also includes somatotroph tumors, lactotroph tumors, mixed somatotroph-lactotroph tumors, and mature/immature PIT1-lineage plurihormonal tumors, whereas gonadotroph tumors are classified within the SF1 lineage. This classification system has created areas of overlap in lineage assignment and nomenclature when cases previously diagnosed mainly on the basis of hormone expression are re-evaluated(1).

All three cases in this cohort expressed PIT1 and PRL and showed variable GH expression, indicating a shared background of PIT1-lineage differentiation. However, cases 2 and 3 also expressed SF1, and case 1 was SF1-negative but LH-positive. These findings indicate that the tumors in this cohort are not uniform, typical PIT1-lineage tumors but instead show variable degrees of plurihormonal or multilineage-marker expression. This feature is central to the present study: in the traditional sense, acidophil stem cell adenoma, mixed GH-PRL adenoma, PIT1-lineage plurihormonal PitNET, and PIT1/SF1 multilineage PitNET may exist along a morphological and immunophenotypic continuum rather than representing completely discrete entities.

### 4.2 Rationale and conservatism of the “acidophil stem cell-like” diagnosis

Classic acidophil stem cell PitNET is an uncommon PIT1-lineage tumor and is generally considered to be related to differentiation from a common progenitor of lactotrophs and somatotrophs. Its typical features include eosinophilic or oncocytic-like cytoplasm, cytoplasmic vacuolation or granularity, predominant PRL expression, weak or focal GH expression, PIT1 positivity, frequent ER expression, and abnormal cytokeratin aggregation or scattered fibrous-body-like patterns. Classic ultrastructural features include dilated or giant mitochondria in the cytoplasm (6).

The present cases share several features supporting acidophil stem cell-like differentiation: all three expressed PIT1 and PRL; all showed focal, patchy, or variable GH expression; CK8 or CK8/18 was expressed in all cases; and none of the patients had typical clinical manifestations of acromegaly, suggesting that GH immunoexpression was not fully concordant with functional GH secretion. ER positivity in cases 2 and 3 was also consistent with PIT1-lineage, particularly lactotroph-related, differentiation.

Nevertheless, the present cases also differed from classic acidophil stem cell adenoma in several respects. Case 1 was ER-negative and LH-positive. Cases 2 and 3 expressed PIT1/PRL/GH but also showed SF1 positivity, whereas FSH and LH were negative. In addition, electron microscopy was not available, and the classic giant mitochondrial change emphasized in acidophil stem cell tumors could not be confirmed. Therefore, the term “acidophil stem cell-like PitNET” rather than a definitive diagnosis of “classic acidophil stem cell PitNET,” more accurately reflects the strength of the current pathological evidence.

### 4.3 PIT1/SF1 lineage-marker co-expression and diagnostic boundaries

The most noteworthy finding in this cohort is the simultaneous expression of SF1 in cases 2 and 3 in the setting of PIT1 positivity, PRL positivity, and variable GH expression. According to the traditional lineage concept, PIT1 and SF1 represent different directions of adenohypophyseal cell differentiation, and their co-expression is readily interpreted as evidence of multilineage PitNET or plurihormonal PitNET. However, recent studies suggest that PIT1/SF1 co-expression may not be incidental, particularly in some somatotroph-related PitNETs, and may represent a special molecular and histological subtype that has not yet been fully recognized. Some studies have noted that the 2022 WHO classification places PitNETs with multi-transcription-factor co-expression within the “plurihormonal” category, although the biological significance of PIT1/SF1 co-expressing tumors requires further refinement (7).

Cases 2 and 3 in this cohort both showed PIT1/SF1 co-expression but were negative for FSH and LH, indicating incomplete evidence of “gonadotroph lineage” differentiation. At least three interpretations are possible: first, the tumor cells may truly co-express PIT1/SF1 multilineage transcription factors; second, the tumor may contain two cellular components, one of PIT1 lineage and one of SF1 lineage, representing a mixed or collision tumor; and third, SF1 positivity may represent aberrant or noncanonical lineage-marker expression rather than mature gonadotropin secretion. In the present study, spatially matched serial-section mapping in cases 2 and 3 demonstrated PIT1-positive and SF1-positive nuclei in morphologically corresponding tumor cells. This finding supports PIT1/SF1 lineage-marker co-expression within the same tumor-cell population. Nevertheless, serial-section mapping is not identical to dual-label immunohistochemistry; therefore, we avoid assigning these tumors to a definitive biological category solely on this basis.

In case 1, the strong and diffuse LH expression, together with spatial correspondence of PIT1 and LH staining in serial sections, makes an interpretation based solely on entrapped non-neoplastic gonadotroph cells less likely. Nevertheless, because SF1 and FSH were negative and serial-section mapping is not equivalent to dual-label immunohistochemistry, the finding is interpreted conservatively as heterogeneous LH lineage-marker expression within a PIT1-lineage PitNET.

### 4.4 Immunophenotypic heterogeneity among the present cases

The three cases illustrate an immunophenotypically heterogeneous spectrum. Case 1 was dominated by PIT1-lineage expression, with PRL positivity, variable GH expression, and concurrent LH positivity but SF1 negativity, placing it closer to the diagnostic range of acidophil stem cell-like PitNET or mixed GH-PRL-related tumors. Cases 2 and 3 showed PIT1, PRL, and variable GH expression with concurrent SF1 positivity, demonstrating PIT1/SF1 lineage-marker co-expression. Together, these cases illustrate heterogeneous lineage-marker expression patterns within acidophil stem cell-like PITNETs.

This heterogeneity has important diagnostic implications. First, it indicates that acidophil stem cell-like PitNET may not fully conform to a textbook immunophenotype, particularly when electron microscopy is lacking; the diagnosis should therefore remain open and evidence-based. Second, it suggests that PIT1/SF1 co-expressing cases may previously have been classified as acidophil stem cell adenoma, mixed GH-PRL adenoma, or plurihormonal adenoma and should be re-examined under the 2022 WHO classification. Third, it indicates that pathology reports should not merely provide a nonspecific diagnosis of “pituitary adenoma” or “plurihormonal adenoma.” Instead, they should describe, as far as possible, the combined pattern of transcription factors, hormones, CK, ER, SSTR2, and other markers, thereby providing a basis for clinical assessment of functional status, invasion risk, and subsequent treatment.

### 4.5 Local invasiveness as an important shared feature

Although the clinical endocrine manifestations in this cohort were atypical, all three cases showed varying degrees of local invasiveness. Case 1 was a giant tumor with bilateral Knosp grade 3 involvement, cavernous sinus involvement, sphenoid sinus and sellar floor bone invasion, and optic chiasm compression; the Ki-67 index was approximately 5%-10%, suggesting a relatively high risk of recurrence or progression. Case 2 showed inferior tumor growth, sellar floor bone invasion, bilateral internal carotid artery encasement, and suspected cerebrospinal fluid rhinorrhea. Although the tumor in case 3 was smaller than that in case 1, intraoperative findings also showed a thin and invaded sellar floor. The clinical relevance of suprasellar extension and compression of adjacent structures has also been emphasized in a large single-center series of suprasellar pituitary adenomas, supporting the need for standardized assessment of optic-chiasm compression, cavernous sinus extension, and surgical risk in tumors with extensive sellar or suprasellar growth (8).

These findings indicate that acidophil stem cell-like PitNET can show clear local invasiveness even when clinically hyposecretory or silent. In particular, tumors with cavernous sinus involvement, internal carotid artery encasement, sellar floor bone destruction, sphenoid-sinus compression with fluid-like signal, or intraoperative cerebrospinal fluid leakage warrant closer postoperative imaging and endocrine follow-up. The Ki-67 index of approximately 5%-10% in case 1 was substantially higher than those in cases 2 and 3, indicating that proliferative activity and invasiveness may not be fully concordant, although proliferative activity remains an important reference for risk assessment.

### 4.6 Differential diagnosis from related PitNET subtypes

The main differential diagnoses in this cohort include the following PitNET subtypes.

First, mixed somatotroph-lactotroph PitNET should be considered. This tumor type usually consists of two components, somatotroph cells and lactotroph cells, and can express both GH and PRL. If GH and PRL are localized to different cell populations and two cellular components are morphologically identifiable, a mixed GH-PRL tumor should be considered. In this cohort, all cases expressed PRL and GH; however, GH expression ranged from substantial expression in case 1 to focal expression in cases 2 and 3; however, no clearly separable GH-positive and PRL-positive tumor-cell populations were identified. Therefore, the findings remain distinct from those of a typical mixed GH-PRL tumor.

Second, mammosomatotroph PitNET should be considered. In this tumor type, the same tumor cells usually co-express GH and PRL, are often ER-positive, and may clinically present as acromegaly with hyperprolactinemia. The present cases lacked typical clinical acromegaly, showed weak or focal GH expression, and cases 2 and 3 were SF1-positive; therefore, they cannot be simply classified as mammosomatotroph tumors.

Third, mature or immature PIT1-lineage plurihormonal PitNETs should be considered. Mature PIT1-lineage plurihormonal tumors usually express PIT1 and may express multiple hormones, including GH, PRL, TSH, and alpha-subunit. Immature PIT1-lineage tumors may show diffuse PIT1 positivity with unstable or incomplete hormone expression. All three cases in this cohort were PIT1-positive but TSH-negative, and cases 2 and 3 were SF1-positive; therefore, the findings cannot be fully explained by PIT1-lineage plurihormonal PitNET alone. Rare monomorphous GH/TSH-secreting adenomas with acidophil stem cell-like ultrastructural features have also been reported, supporting the concept of overlap between plurihormonal PIT1-lineage tumors and acidophil stem cell-like differentiation (9). A single-center series of TSH/GH cosecreting pituitary adenomas further illustrates the clinical and biochemical heterogeneity of PIT1-lineage plurihormonal tumors (10). However, because TSH was negative in all three present cases, a TSH/GH cosecreting PitNET is not favored in the current cohort.

Fourth, gonadotroph PitNET or PIT1/SF1 multilineage PitNET should be considered. Cases 2 and 3 expressed SF1, but FSH and LH were negative, indicating limited evidence of gonadotroph differentiation. In the present cases, spatially matched serial sections support PIT1/SF1 co-expression. Future dual-label immunostaining and molecular studies may further clarify whether this pattern represents a distinct biological subgroup.

### 4.7 Implications for pathological diagnosis and reporting

This cohort suggests that, when encountering a pituitary tumor that is PIT1-positive and PRL-positive and shows weak, focal, or otherwise variable GH expression, pathologists should not directly diagnose mixed GH-PRL adenoma or plurihormonal adenoma solely on the basis of “PRL + GH.” Instead, the following factors should be further evaluated: (1) the combination of PIT1, SF1, and TPIT transcription factors; (2) the extent and intensity of PRL, GH, TSH, FSH, LH, and ACTH expression; (3) the localization pattern of CK8/18 or CAM5.2, particularly whether perinuclear dot-like or fibrous-body-like structures are present (11); (4) auxiliary markers such as ER and alpha-subunit; and (5) radiological invasiveness, intraoperative findings, and clinical hormone levels.

For the present cases, a more prudent reporting strategy may include two levels. The first level is the definitive diagnosis: PitNET with PIT1-lineage differentiation, PRL expression and variable GH expression; the morphology and immunophenotype are consistent with acidophil stem cell-like differentiation. The second level is a diagnostic comment: in some cases, SF1 or LH expression is present, indicating multilineage/plurihormonal expression; the relationship with PIT1/SF1 multilineage PitNET should be described as PIT1/SF1 lineage-marker co-expression, supported in the present study by spatially matched serial-section mapping. Dual-label immunostaining and molecular testing may provide further confirmation in future studies. This reporting approach reflects the conceptual framework of the 2022 WHO classification while avoiding overly precise classification when the available evidence is insufficient. Based on this two-level reporting strategy, **Table 2** summarizes a reporting-oriented, case-by-case interpretation of the diagnostic boundaries in the present cohort.

**Table 2:** Integrated reporting-oriented diagnostic interpretation and diagnostic-boundary considerations.

Feature	Case 1	Case 2	Case 3
Working integrated diagnosis	Acidophil stem cell-like PitNET with PIT1-lineage differentiation and heterogeneous LH lineage-marker expression (PIT1 100%, PRL 90%, GH 50%, LH 90%; SF1 and FSH negative).	Acidophil stem cell-like PitNET with spatially supported PIT1/SF1 lineage-marker co-expression (PIT1, SF1, and PRL each 100%; GH 10%; FSH and LH negative).	Acidophil stem cell-like PitNET with spatially supported PIT1/SF1 lineage-marker co-expression (PIT1, SF1, and PRL each 100%; GH 10%; FSH and LH negative).
Key morphology and ancillary immunophenotype	Eosinophilic/granular cytoplasm with focal pale, chromophobic, or vacuolated change; CK8 2% membranous; ER negative; SSTR2 90% strong membranous; Ki-67 approximately 5%-10%.	Original pathology report described a plurihormonal adenoma with acidophil- and gonadotroph-lineage-marker expression; CK8/18 80% paranuclear dot-like; ER 60% moderate nuclear; SSTR2 negative; Ki-67 approximately 1%.	Original pathology report described a plurihormonal adenoma with gonadotroph- and acidophil-lineage-marker expression; CK8/18 100% strong membranous; ER 100% strong nuclear; SSTR2 negative; Ki-67 approximately 2%.
Spatial relationship of PIT1 and SF1	SF1 negative (0%); therefore, a PIT1/SF1 co-expression question does not arise in this case.	Spatially matched serial sections demonstrated PIT1-positive and SF1-positive nuclei in morphologically corresponding tumor cells (**Supplementary Figure 1**), supporting intratumoral PIT1/SF1 lineage-marker co-expression.	Spatially matched serial sections demonstrated PIT1-positive and SF1-positive nuclei in morphologically corresponding tumor cells (**Supplementary Figure 1**), supporting intratumoral PIT1/SF1 lineage-marker co-expression.
Interpretation of heterogeneous lineage-marker expression	Diffuse LH expression in a PIT1-positive/SF1-negative tumor is best reported as heterogeneous LH lineage-marker expression. LH expression alone does not establish a separately functional gonadotroph phenotype.	Diffuse SF1 expression with absent FSH and LH supports a heterogeneous transcription-factor profile rather than a clinically functional gonadotroph PitNET.	Diffuse SF1 expression with absent FSH and LH supports a heterogeneous transcription-factor profile rather than a clinically functional gonadotroph PitNET.
Principal diagnostic boundary	Acidophil stem cell-like PitNET versus mixed somatotroph-lactotroph PitNET versus mature PIT1-lineage plurihormonal PitNET with additional LH expression.	Acidophil stem cell-like PitNET versus PIT1-lineage plurihormonal PitNET with heterogeneous PIT1/SF1 lineage-marker co-expression. A simple spatially separate collision or double PitNET is less likely, although it cannot be definitively excluded without dual-label immunohistochemistry or molecular analysis.	Acidophil stem cell-like PitNET versus PIT1-lineage plurihormonal PitNET with heterogeneous PIT1/SF1 lineage-marker co-expression. A simple spatially separate collision or double PitNET is less likely, although it cannot be definitively excluded without dual-label immunohistochemistry or molecular analysis.
Why a classic acidophil stem cell PitNET designation remains cautious	Electron microscopy was not performed. The comparatively extensive GH (50%) and LH (90%) expression extends beyond a purely PRL-predominant profile.	Electron microscopy was not performed. Diffuse SF1 expression and focal GH (10%) expression extend beyond a conventional acidophil stem cell phenotype.	Electron microscopy was not performed. Diffuse SF1 expression and focal GH (10%) expression extend beyond a conventional acidophil stem cell phenotype.
Risk-relevant clinical and radiological context	Giant invasive tumor; bilateral Knosp grade 3; cavernous sinus, sellar-floor, and sphenoid sinus involvement; Ki-67 approximately 5%-10%; no radiological recurrence at 14.7 months.	Knosp grade 4; bilateral internal carotid artery encasement, sellar-floor involvement, and suspected CSF rhinorrhea; Ki-67 approximately 1%; no radiological recurrence at 51.3 months.	Knosp grade 1; right parasellar/cavernous extension and sellar-floor involvement; Ki-67 approximately 2%; no radiological recurrence at 57.6 months.
Suggested final diagnostic comment/manuscript wording	PitNET with PIT1-lineage differentiation, diffuse PRL expression and substantial GH expression, and diffuse LH expression; morphology is consistent with acidophil stem cell-like differentiation. In the absence of SF1 and FSH, LH should be reported as heterogeneous lineage-marker expression rather than independent gonadotroph differentiation.	PitNET with PIT1-lineage differentiation, diffuse PRL/PIT1/SF1 expression, and focal GH expression; morphology is consistent with acidophil stem cell-like differentiation. Spatially matched serial sections support PIT1/SF1 lineage-marker co-expression in morphologically corresponding tumor cells. FSH and LH are negative.	PitNET with PIT1-lineage differentiation, diffuse PRL/PIT1/SF1 expression, and focal GH expression; morphology is consistent with acidophil stem cell-like differentiation. Spatially matched serial sections support PIT1/SF1 lineage-marker co-expression in morphologically corresponding tumor cells. FSH and LH are negative.
Additional studies that could refine classification	Electron microscopy for giant mitochondria; alpha-subunit and D2R/SSTR5 testing if material is available and clinically relevant.	Electron microscopy and molecular testing may refine classification. Dual-label PIT1/SF1 immunohistochemistry could provide orthogonal confirmation of the serial-section result if tissue remains available.	Electron microscopy and molecular testing may refine classification. Dual-label PIT1/SF1 immunohistochemistry could provide orthogonal confirmation of the serial-section result if tissue remains available.

### 4.8 Therapeutic and follow-up implications

Clinical management of these cases should emphasize two aspects. First, a hyposecretory or silent phenotype does not imply low risk. Case 1 showed giant invasive growth with an elevated Ki-67 index. Although the proliferation indices in cases 2 and 3 were not high, both cases showed local invasive features such as sellar floor bone invasion or internal carotid artery encasement. Therefore, long-term postoperative imaging follow-up is required, with dynamic monitoring of PRL, GH, IGF-1, and pituitary function (12).

Second, heterogeneity of receptor expression may be relevant to treatment selection in residual, recurrent, or progressive disease (13). SSTR2 expression differed among the present cases: case 1 showed strong membranous positivity, whereas cases 2 and 3 were negative. These findings indicate that even among acidophil stem cell-like or PIT1-related tumors, somatostatin-receptor expression may vary. For residual, recurrent, or progressive cases, additional testing for SSTR5 and D2R may provide useful information for treatment with somatostatin analogues, dopamine receptor agonists, or other combined therapies (14).

### 4.9 Limitations

This study has several limitations. First, the number of cases is small, and the study is retrospective; therefore, the true incidence and prognostic differences of this tumor type cannot be assessed. Second, electron microscopy was not performed, and the classic giant mitochondrial feature of acidophil stem cell tumors could not be confirmed. Third, IGF-1 was not measured in any case, and the available GH values were obtained at limited time points. Therefore, biochemical assessment of GH excess was incomplete.

Future studies should expand the case number, standardize review of hematoxylin-eosin morphology and immunohistochemistry, supplement markers such as alpha-subunit, SSTR5, and D2R, and perform dual-label immunohistochemistry, molecular testing, or methylation analysis whenever possible. These approaches may further clarify the relationship between acidophil stem cell-like PitNET and PIT1/SF1 multilineage PitNET.

### 4.10 Conclusion

In summary, these three cases suggest that acidophil stem cell-like PitNETs display substantial immunophenotypic heterogeneity within the framework of the 2022 WHO classification. In addition to PIT1, PRL, and variable GH expression, some cases may also express gonadotroph-associated markers, including SF1 or LH, raising diagnostic-boundary questions at the interface with PIT1-lineage plurihormonal PitNETs and PIT1/SF1 co-expressing tumors. These tumors may present as clinically hyposecretory or silent lesions but can still show local invasiveness. Therefore, pathological diagnosis and risk assessment should integrate transcription factors, hormone expression, CK pattern, ER/SSTR2 status, radiological invasiveness, and long-term follow-up results, rather than relying solely on a single hormone marker or clinical endocrine manifestation.

## Data Availability

The original contributions presented in the study are included in the article/**Supplementary Material**. Further inquiries can be directed to the corresponding author.
