# Supplementary material for: Acidophil stem cell-like pituitary neuroendocrine tumors with heterogeneous PIT1/SF1 or LH lineage-marker expression: a clinicopathological study of three cases
Source: Front Endocrinol (Lausanne). 2026 Jul 23;17:1879290. doi: 10.3389/fendo.2026.1879290 (PMC13441817; doi:10.3389/fendo.2026.1879290)
Supplement: Supplementary file 1 [file Table1.docx]

**Supplementary Tables**

**Supplementary Table 1. Detailed clinicoradiological, surgical, pathological, and follow-up characteristics of the three cases.**

| **Feature** | **Case 1** | **Case 2** | **Case 3** |
| --- | --- | --- | --- |
| **Patient / institution / surgery year** | Male, 17 years; Shandong University Qilu Hospital Dezhou Hospital; 2024. | Male, 34 years; Yantai Yuhuangding Hospital Affiliated to Qingdao University; 2021. | Male, 48 years; Yantai Yuhuangding Hospital Affiliated to Qingdao University; 2021. |
| **Presentation and clinical endocrine phenotype** | Progressive visual impairment and left temporal visual-field defect for >6 months. No lip/tongue enlargement, acral enlargement, polydipsia, or polyuria; no definite clinical acromegaly. | Pituitary mass detected 1 month earlier; watery to lightly blood-stained rhinorrhea for 5 days, aggravated by bending/head lowering; CSF rhinorrhea was suspected. No documented acromegaly. | Headache for 2 months, aggravated for 1 week, with nausea/vomiting. No hyposexuality, polydipsia/polyuria, acromegaly, body-weight change, or centripetal obesity. |
| **Preoperative endocrine overview** | PRL 30.82 ng/mL; GH 1.336 ng/mL; IGF-1 not measured. Baseline gonadal-axis values were available; detailed values and reference ranges are shown in Supplementary Table 2. | Initial PRL >470.0 ng/mL before surgery; bromocriptine was administered preoperatively. IGF-1 not measured. Detailed values and reference ranges are shown in Supplementary Table 2. | PRL 504.9 ng/mL; GH 0.186 ng/mL; IGF-1 not measured. Detailed values and reference ranges are shown in Supplementary Table 2. |
| **Preoperative MRI and Knosp grade** | Sellar/suprasellar mass, approximately 6.0 x 4.5 x 3.8 cm; bilateral Knosp grade 3 involvement. | Initial MRI (February 2021): approximately 2.5 x 2.1 x 3.4 cm. Preoperative repeat MRI (27 March 2021, after bromocriptine): approximately 2.1 x 1.9 x 2.9 cm; maximum Knosp grade 4. | Intrasellar lesion, approximately 2.6 x 1.6 x 2.0 cm; Knosp grade 1. |
| **Key invasive / compressive features** | Suprasellar extension with optic-chiasm compression; bilateral cavernous sinus extension; sellar-floor erosion and extension into the sphenoid sinus; adhesion/encasement involving the internal carotid arteries. | Inferior extension with sellar-floor depression/thinning and tumor involvement; sphenoid-sinus compression with fluid-like signal; bilateral ICA encasement, more prominent on the left; no optic-chiasm compression on MRI. | Right parasellar/cavernous sinus extension consistent with Knosp grade 1; optic chiasm elevated/compressed; sellar floor thin and involved intraoperatively. |
| **Surgery and key intraoperative findings** | 17 December 2024; endoscopic transnasal/transsphenoidal resection. Tumor protruded into the sphenoid sinus and invaded the sellar floor; bilateral cavernous-sinus tumor was largely removed. High-flow CSF leak occurred and was repaired with fascia lata/fat graft and a vascularized flap. | 2 April 2021; navigation-guided endoscopic transnasal/transsphenoidal resection. Sellar floor was thin and invaded. Tumor was friable/fish-flesh-like, relatively vascular, with abundant fibrous strands/septa; approximately 3 x 3 x 2 cm was removed. No obvious intraoperative CSF leak. | 22 January 2021; navigation-assisted endoscopic transnasal/transsphenoidal resection with CT navigation. Sellar floor was thin and invaded. Gray-white, relatively vascular intrasellar tumor was aspirated/removed; operative estimate approximately 3 x 3 x 2 cm. |
| **Postoperative course and endocrine management** | Postoperative agitation and fever; high-flow CSF leak managed with lumbar cistern drainage and anti-infective treatment. Visual acuity improved. Prednisone 5 mg twice daily for 2 weeks was prescribed at discharge because pituitary hormone levels were considered low. | No persistent nasal/nasopharyngeal leakage after surgery; treated for fever/inflammatory CSF changes and discharged in stable condition. No postoperative endocrine replacement therapy was documented in the supplied record. | No documented postoperative CSF rhinorrhea, obvious polydipsia/polyuria, or major electrolyte abnormality; discharged in stable condition. No postoperative endocrine replacement therapy was documented in the supplied record. |
| **Integrated pathological summary (updated Tables 1-2)** | Acidophil stem cell-like PitNET morphology with PIT1-lineage differentiation and heterogeneous LH lineage-marker expression: PIT1 100%, PRL 90%, GH 50%, LH 90%; SF1, FSH and TPIT negative. CK8/18 2% membranous; ER negative; SSTR2 90% strong membranous; Ki-67 approximately 5%-10%. | Acidophil stem cell-like PitNET with spatially supported PIT1/SF1 lineage-marker co-expression: PIT1, SF1 and PRL each 100%; GH 10%; FSH, LH and TPIT negative. CK8/18 80% paranuclear dot-like; ER 60% moderate nuclear; SSTR2 negative; Ki-67 approximately 1%. | Acidophil stem cell-like PitNET with spatially supported PIT1/SF1 lineage-marker co-expression: PIT1, SF1 and PRL each 100%; GH 10%; FSH, LH and TPIT negative. CK8/18 100% strong membranous; ER 100% strong nuclear; SSTR2 negative; Ki-67 approximately 2%. |
| **Spatial assessment of PIT1/SF1 expression** | Not applicable because SF1 was negative (0%). | Spatially matched serial sections demonstrated PIT1-positive and SF1-positive nuclei in morphologically corresponding tumor cells, supporting PIT1/SF1 lineage-marker co-expression (Supplementary Figure 1). | Spatially matched serial sections demonstrated PIT1-positive and SF1-positive nuclei in morphologically corresponding tumor cells, supporting PIT1/SF1 lineage-marker co-expression (Supplementary Figure 1). |
| **Early postoperative MRI / residual tumor status** | No visible residual tumor; gross total resection reported on early postoperative MRI. | No visible residual tumor; gross total resection reported on early postoperative MRI. | No visible residual tumor; gross total resection reported on early postoperative MRI. |
| **Last MRI and imaging follow-up duration** | 8 March 2026: no radiological recurrence or progression; 446 days (14.7 months) after surgery. | 12 July 2025: no radiological recurrence or progression; 1,562 days (51.3 months) after surgery. | 10 November 2025: no radiological recurrence or progression; 1,753 days (57.6 months) after surgery. |
| **Follow-up modality** | Repeat serum hormone testing and sellar MRI. | Repeat serum hormone testing and sellar MRI. | Repeat serum hormone testing and sellar MRI. |

*Abbreviations: CSF, cerebrospinal fluid; GH, growth hormone; ICA, internal carotid artery; IGF-1, insulin-like growth factor 1; MRI, magnetic resonance imaging; PRL, prolactin; PitNET, pituitary neuroendocrine tumor; SSTR2, somatostatin receptor subtype 2.*

*Note: In cases 2 and 3, PIT1/SF1 co-expression was assessed by spatially matched serial-section mapping in morphologically corresponding tumor cells and was not evaluated by dual-label immunohistochemistry.*

**Supplementary Table 2. Detailed preoperative, early postoperative, and last available endocrine data for the three cases.**

*Surgery dates: Case 1, 17 December 2024; Case 2, 2 April 2021; Case 3, 22 January 2021.*

**Case 1**

| **Hormone / axis** | **Preoperative value(s) (date: value)** | **Early postoperative value(s) (date: value)** | **Lastest available follow-up value (date: value)** | **Unit** | **Laboratory reference range / note** |
| --- | --- | --- | --- | --- | --- |
| **PRL** | 2024-12-12: 30.82 | 2024-12-18: 2.66 | 2026-03-08: 6.53 | ng/mL | 2.64-13.13 |
| **GH** | 2024-12-12: 1.336 | 2024-12-20: 1.622 | 2026-03-08: 0.650 | ng/mL | 0.003-0.971 |
| **IGF-1** | NM | NM | NM | - | Not available; not measured |
| **ACTH** | NA | NA | 2026-03-08: 1.09 | pg/mL | 7.2-63.4 |
| **Cortisol** | 2024-12-12: 4.26 | 2024-12-18: 3.14 | 2026-03-08: 0.08 | µg/dL | 0-22.6 |
| **TSH** | 2024-12-12: 5.022 | 2024-12-20: 1.907 | 2026-03-08: 10.862 | mIU/mL | 0.56-5.91 |
| **FT4** | 2024-12-12: 24.53 | 2024-12-20: 6.71 | 2026-03-08: 26.87 | pg/mL | 6.2-12.4 |
| **FT3** | 2024-12-12: 9.72 | 2024-12-20: 3.10 | 2026-03-08: 8.01 | pg/mL | 2.3-4.8 |
| **LH** | 2024-12-12: 4.47 | 2024-12-18: 0.71 | 2025-01-20: 0.37 | mIU/mL | Source displayed lower limit only: >=1.24 |
| **FSH** | 2024-12-12: 6.25 | 2024-12-18: 3.34 | 2025-01-20: 0.58 | mIU/mL | Source displayed lower limit only: >=1.27 |
| **Testosterone** | 2024-12-12: 2.20 | 2024-12-18: 0.46 | 2026-03-08: 0.54 | ng/mL | 1.75-7.81 |
| **Estradiol** | 2024-12-12: 19.45 | 2024-12-18: 15.04 | 2025-01-20: 10.00 | pg/mL | Source displayed lower limit only: >=15 |
| **Progesterone** | 2024-12-12: 0.32 | 2024-12-18: 0.16 | 2025-01-20: 0.10 | ng/mL | Source displayed lower limit only: >=0.14 |

*Case 1 note: The early postoperative value is the first available measurement after surgery; the last available follow-up value is shown for each analyte and may have been obtained at different follow-up times.*

**Case 2**

| **Hormone / axis** | **Preoperative value(s) (date: value)** | **Early postoperative value(s) (date: value)** | **Lastest available follow-up value (date: value)** | **Unit** | **Laboratory reference range / note** |
| --- | --- | --- | --- | --- | --- |
| **PRL** | 2021-02-21: >470.0; 2021-02-28: >470.0 | 2021-04-03: 294.8 | 2025-07-12: 21.49 | ng/mL | 4.6-21.4 (2021-2024); 4.04-15.20 (2025). Bromocriptine was administered preoperatively; 2021-03-29 PRL 111.6 ng/mL was obtained after treatment. |
| **GH** | 2021-02-28: 1.15; 2021-03-30: 0.528 | 2021-04-03: 1.77 | 2021-04-03: 1.77 | ng/mL | 0.03-2.47 |
| **IGF-1** | NM | NM | NM | - | Not available; not measured |
| **ACTH** | 2021-02-28: 32.01; 2021-03-30: 21.17 | 2021-04-03: 16.82 | 2021-04-03: 16.82 | pg/mL | 7.2-63.6 |
| **Cortisol (labeled 8 h)** | 2021-02-28: 189.2; 2021-03-30: 279.2 | 2021-04-03: 434.7 | 2021-04-03: 434.7 | nmol/L | 172.0-497.0 |
| **Cortisol (labeled 16 h)** | 2021-02-28: 210.0; 2021-03-30: 376.4 | 2021-04-04: 193.6 | 2021-04-04: 193.6 | nmol/L | 74.1-286.0 |
| **TSH** | 2021-02-28: 3.10; 2021-03-30: 2.07 | 2021-04-03: 2.76 | 2021-04-03: 2.76 | mIU/L | 0.27-4.20 |
| **FT4** | 2021-02-28: 12.24; 2021-03-30: 14.92 | 2021-04-03: 21.31 | 2021-04-03: 21.31 | pmol/L | 12.00-22.00 |
| **FT3** | 2021-02-28: 4.05; 2021-03-30: 3.84 | 2021-04-03: 4.01 | 2021-04-03: 4.01 | pmol/L | 2.8-7.1 |
| **LH** | 2021-02-21: 1.73; 2021-02-28: 1.16 | 2021-04-03: 2.99 | 2024-08-10: 3.04 | mIU/mL | 1.7-8.6 |
| **FSH** | 2021-02-21: 2.93; 2021-02-28: 2.77 | 2021-04-03: 3.03 | 2024-08-10: 4.16 | mIU/mL | 1.5-12.4 |
| **Testosterone** | 2021-02-21: 1.10; 2021-02-28: 1.11 | 2021-04-03: 1.88 | 2024-08-10: 2.21 | ng/mL | 2.49-8.36 |
| **Estradiol** | 2021-02-21: 24.20; 2021-02-28: 16.67 | 2021-04-03: 21.85 | 2024-08-10: 11.64 | pg/mL | NR in source |
| **Progesterone** | 2021-02-21: 0.299; 2021-02-28: 0.271 | 2021-04-03: 0.715 | 2024-08-10: <0.050 | ng/mL | 0.00-0.149; latest assay reported <0.149 |

*Case 2 note: The initial preoperative PRL value was above the assay upper-reporting limit. Bromocriptine was administered before surgery; the PRL value of 111.6 ng/mL on 29 March 2021 was obtained after treatment and is not used as the baseline preoperative value.*

**Case 3**

| **Hormone / axis** | **Preoperative value(s) (date: value)** | **Early postoperative value(s) (date: value)** | **Lastest available follow-up value (date: value)** | **Unit** | **Laboratory reference range / note** |
| --- | --- | --- | --- | --- | --- |
| **PRL** | 2021-01-20: 504.9 | 2021-01-23: 25.12 | 2025-11-11: 16.0 | ng/mL | 4.6-21.4 (2021-2023); 4.04-15.20 (2025) |
| **GH** | 2021-01-20: 0.186 | NA | 2023-05-08: 0.060 | ng/mL | 0.03-2.47 |
| **IGF-1** | NM | NM | NM | - | Not available; not measured |
| **ACTH** | 2021-01-20: 26.51 | 2021-01-23: 49.07 | 2021-01-23: 49.07 | pg/mL | 7.2-63.6 |
| **Cortisol (labeled 8 h)** | 2021-01-20: 150.6 | 2021-01-23: 791.1 | 2022-12-07: 151.5 | nmol/L | 172.0-497.0 |
| **Cortisol (labeled 0 h)** | 2021-01-20: 38.23 | NA | NA | nmol/L | NR in source |
| **TSH** | 2021-01-20: 2.19 | 2021-01-23: 1.31 | 2025-11-11: 3.80 | mIU/L | 0.27-4.20 |
| **FT4** | 2021-01-20: 13.92 | 2021-01-23: 15.01 | 2025-11-11: 13.28 | pmol/L | 12.00-22.00 |
| **FT3** | 2021-01-20: 4.96 | 2021-01-23: 3.80 | 2025-11-11: 5.07 | pmol/L | 2.8-7.1 (through 2023); 3.10-6.80 (2025) |
| **LH** | 2021-01-20: 1.98 | 2021-01-23: 4.10 | 2025-11-11: 1.83 | mIU/mL | 1.7-8.6 |
| **FSH** | 2021-01-20: 4.08 | 2021-01-23: 3.86 | 2025-11-11: 3.87 | mIU/mL | 1.5-12.4 |
| **Testosterone** | 2021-01-20: 4.08 | 2021-01-23: 1.85 | 2025-11-11: 3.35 | ng/mL | 2.49-8.36 (through 2023); 1.93-7.40 (2025) |
| **Estradiol** | 2021-01-20: 25.04 | 2021-01-23: 26.96 | 2025-11-11: 12.1 | pg/mL | NR in source |
| **Progesterone** | 2021-01-20: 0.181 | 2021-01-23: 1.02 | 2025-11-11: 0.181 | ng/mL | 0.00-0.149; latest assay reported <0.149 |

*General notes: NM, not measured; NA, no measurement available in the supplied record; NR, laboratory reference range was not displayed in the supplied record. IGF-1 was not measured in any case. Reference ranges are reproduced as reported; some analytes were measured with different assays during follow-up.*
